# Tamoxifen triggers a transcriptional switch from proliferation to differentiation in the circumvallate taste epithelium in mice

**DOI:** 10.1038/s41598-025-32701-8

**Published:** 2025-12-17

**Authors:** Norihito Oura, Eriko Koyanagi-Matsumura, Aya Hagimoto, Mitsuru Saito, Hideto Saijo, Hirohito Miura

**Affiliations:** 1 Oral and Maxillofacial Surgery, Kagoshima University Graduate School of Medical and Dental Sciences, 8-35-1 Sakuragaoka, Kagoshima-shi, Kagoshima 890-8544 Japan; 2 Oral physiology, Kagoshima University Graduate School of Medical and Dental Sciences, Kagoshima-shi, Japan; 3 Maxillofacial Radiology, Kagoshima University Graduate School of Medical and Dental Sciences, Kagoshima-shi, Japan

**Keywords:** Cancer, Cell biology, Developmental biology, Molecular biology

## Abstract

The tamoxifen-inducible Cre-loxP system is an indispensable experimental tool in life sciences for inducing spatiotemporally controlled genetic recombination in the target tissues of living animals. The use of this technology is expected to increase in taste research. However, the direct effects of tamoxifen on taste buds remain largely unexplored. Here, we demonstrate that tamoxifen reduces cell supply to the taste buds in a dose-dependent manner. RNA sequencing of the circumvallate epithelium revealed that tamoxifen induced a transcriptional shift from proliferation to differentiation. The genes regulating the cell cycle were downregulated, whereas genes promoting the differentiation of epithelial cells and keratinocytes were upregulated. Within taste buds, *Shh* was downregulated in immature precursor cells, whereas cell type-specific genes were broadly upregulated in mature taste bud cells. Notably, transcription factors driving taste cell type differentiation, such as *Pou2f3*,* Ascl1*, and *Nkx2-2*, were induced, suggesting that tamoxifen activates transcription to promote the differentiation of all cell types in taste buds, rather than activating particular signaling pathways in specific cell types. These findings indicate that tamoxifen rapidly triggers a transcriptional switch from proliferation to differentiation in the circumvallate taste epithelium, highlighting a potential confounding effect in taste research that employs tamoxifen administration.

**Supplementary Information:**

The online version contains supplementary material available at 10.1038/s41598-025-32701-8.

## Introduction

Gustation plays a crucial role in animal survival by serving as a gatekeeper for ingestion, enabling the detection of appetitive cues from nutritious food sources and aversive cues from potentially harmful items such as toxins. The sensory end organ for gustation, taste buds, is typically located in three types of taste papillae (fungiform, foliate, and circumvallate) and on the soft palate in the oral cavity. Taste buds are of epithelial origin and are maintained by continuous cell renewal originating from bipotential stem cells that give rise to both taste buds and the surrounding epithelium^1,2^. Taste bud cells are classified into four cell types (Type I-IV). Among them, Type IV cells are basal cells that express *Sonic hedgehog* (*Shh*) within the taste buds and are postmitotic precursor cells of Type I-III mature cells^3,4^. *Shh* expression is downregulated during the differentiation of Type I-III cells.

Recently, progress has accelerated in elucidating the molecular and cellular mechanisms of taste bud maintenance using advanced molecular approaches, such as RNA sequencing^5–8^. With increasing molecular knowledge, the importance of precisely examining the in vivo functions of identified genes and properties of cells expressing a particular gene has increased. The tamoxifen-inducible Cre-loxP system is a key technology for this purpose, in which tamoxifen administration induces spatiotemporally controlled genetic recombination in the target tissues of living animals^9^. This system has been employed in various taste bud studies, including lineage tracing and characterization of stem and progenitor cells of taste buds^4,10,11^, functional analysis of *Shh* in taste buds^12,13^, and lineage tracing of Type II and III cell differentiation^14^, and its applications are expected to expand further in taste bud research.

Tamoxifen is the most commonly used drug for patients with estrogen receptor (ER)-positive breast cancer and was initially believed to act through antagonistic mechanisms in the ER. However, it is now recognized as a selective estrogen receptor modulator that can interact with ERs either antagonistically or agonistically, depending on the target tissue and physiological context. Moreover, several studies have indicated that tamoxifen exerts diverse biological effects, including the induction of cell cycle arrest, suppression of mitochondrial respiration, and promotion of apoptosis via ER-independent mechanisms^15^.

Consequently, the direct effects of tamoxifen on target tissues may confound experimental results. In the pancreas, tamoxifen affects cell proliferation in a cell type-specific manner, inhibiting β-cell proliferation and increasing acinar cell proliferation^16^. The effects of tamoxifen on bones are further complicated, with either retardation or promotion of bone growth depending on the bone category and the administration protocol. Although this knowledge may dampen the usability of tamoxifen to some extent, the tamoxifen-inducible Cre-loxP system remains an irreplaceable tool for conditional gene recombination because of its efficiency and accuracy compared with other techniques^17^. Therefore, clarifying the direct effects of tamoxifen on target tissues is essential to minimize confounding influences and ensure the reliability and accuracy of experiments. However, the direct effects of tamoxifen on taste buds remain largely unexplored.

In this study, we investigated the effects of a single intraperitoneal tamoxifen injection on the circumvallate taste epithelium. Unexpectedly, tamoxifen suppressed cell proliferation while promoting the expression of cell maturation-associated genes both within and surrounding the taste buds. These findings suggest that tamoxifen may be a confounding factor in taste research that employs tamoxifen administration and indicate that tamoxifen triggers a transcriptional switch from proliferation to differentiation both within and around taste buds.

## Results

### Tamoxifen upregulates Type I, II, and III markers but downregulates Type IV marker in circumvallate papillae

**Fig. 1:**
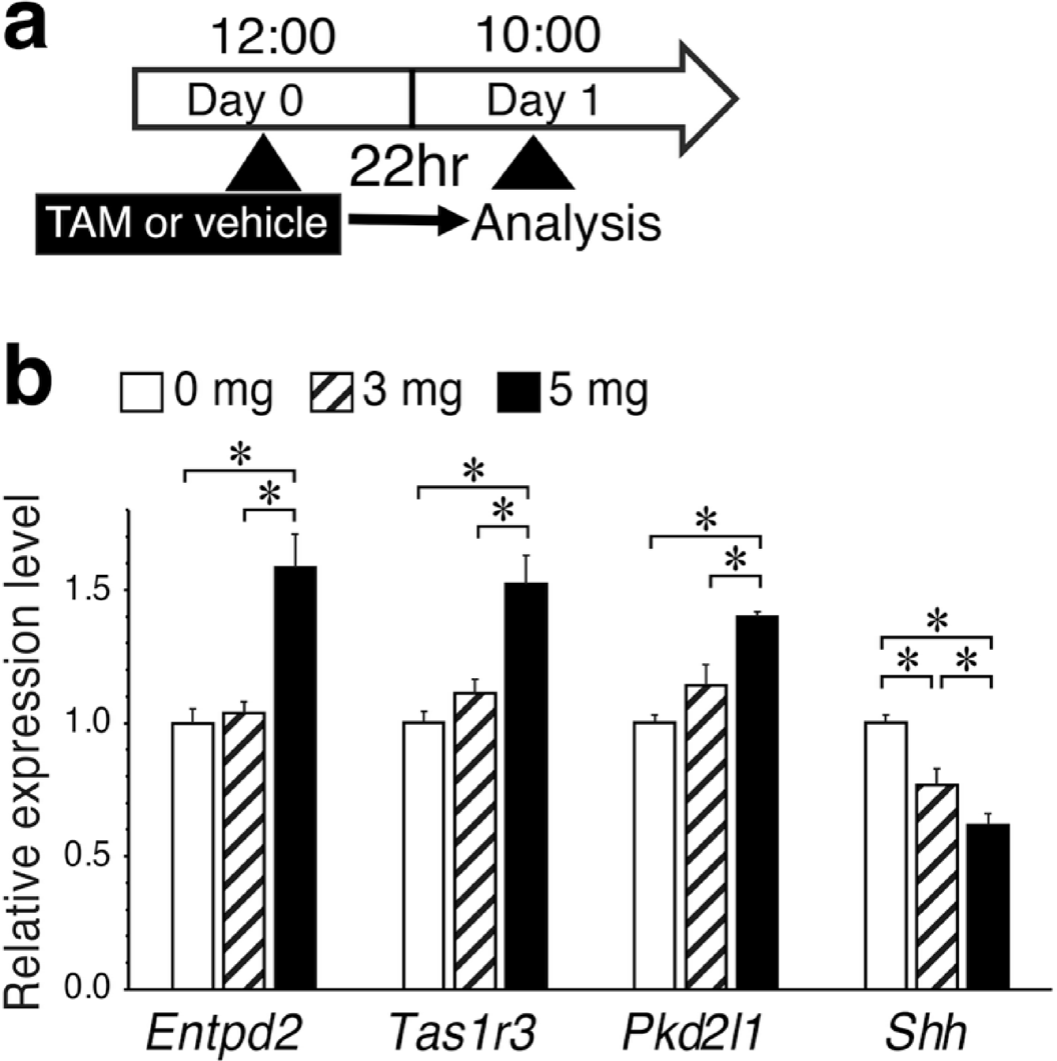
Effect of tamoxifen on the expression of taste cell-type markers in the CV. Quantitative RT-PCR analysis was performed 22 hours after tamoxifen injection. (**a**) Schedule of tamoxifen administration and collection of the CV epithelium. (**b**) Relative mRNA expression levels were normalized to the internal control gene *β-actin* and expressed as ratios relative to the control group (0 mg tamoxifen), with control values set to 1.0. *Entpd2*, *Tas1r3*, *Pkd2l1*, and *Shh* are specific markers of Type I, II, III, and IV cells, respectively. Data are presented as mean ± SD (*n*=4). Asterisks indicate statistically significant differences (ANOVA *p* < 0.05; post-hoc Bonferroni test *p*<0.05).

To assess the effect of tamoxifen on taste buds, we first analyzed the impact of a single intraperitoneal injection on the expression of taste bud cell type markers in the circumvallate papillae (CV) one day after injection (Fig. 1a). Tamoxifen was injected at doses of 0, 3, and 5 mg per 20 g body weight (b.w.), and the expression levels of Type I, II, III, and IV cell markers (*Entpd2*, *Tas1r3*, *Pkd2l1*, and *Shh*, respectively) were analyzed using quantitative RT-PCR (qPCR) (Fig. 1b). The markers for Type I, II, and III mature cells increased in a dose-dependent manner following tamoxifen injection and reached more than 1.4-fold higher levels with the 5 mg dose compared to the control. In contrast, the Type IV immature cell marker, *Shh*, was downregulated. *Shh* expression decreased to nearly 0.6-fold of the control level at the 5 mg dose. These results indicate that tamoxifen upregulates gene expression in mature cell types but downregulates it in immature precursor cells.

### Tamoxifen does not alter cell type marker specificity or increase the number of Type II cells

The tamoxifen-induced increase in the expression of Type I, II, and III cell markers prompted us to examine two possibilities: (1) whether tamoxifen caused misexpression of cell type-specific markers, leading to crossover between cell types, thereby increasing overall expression, or (2) whether it increased the number of cells in each cell type.

**Fig. 2:**
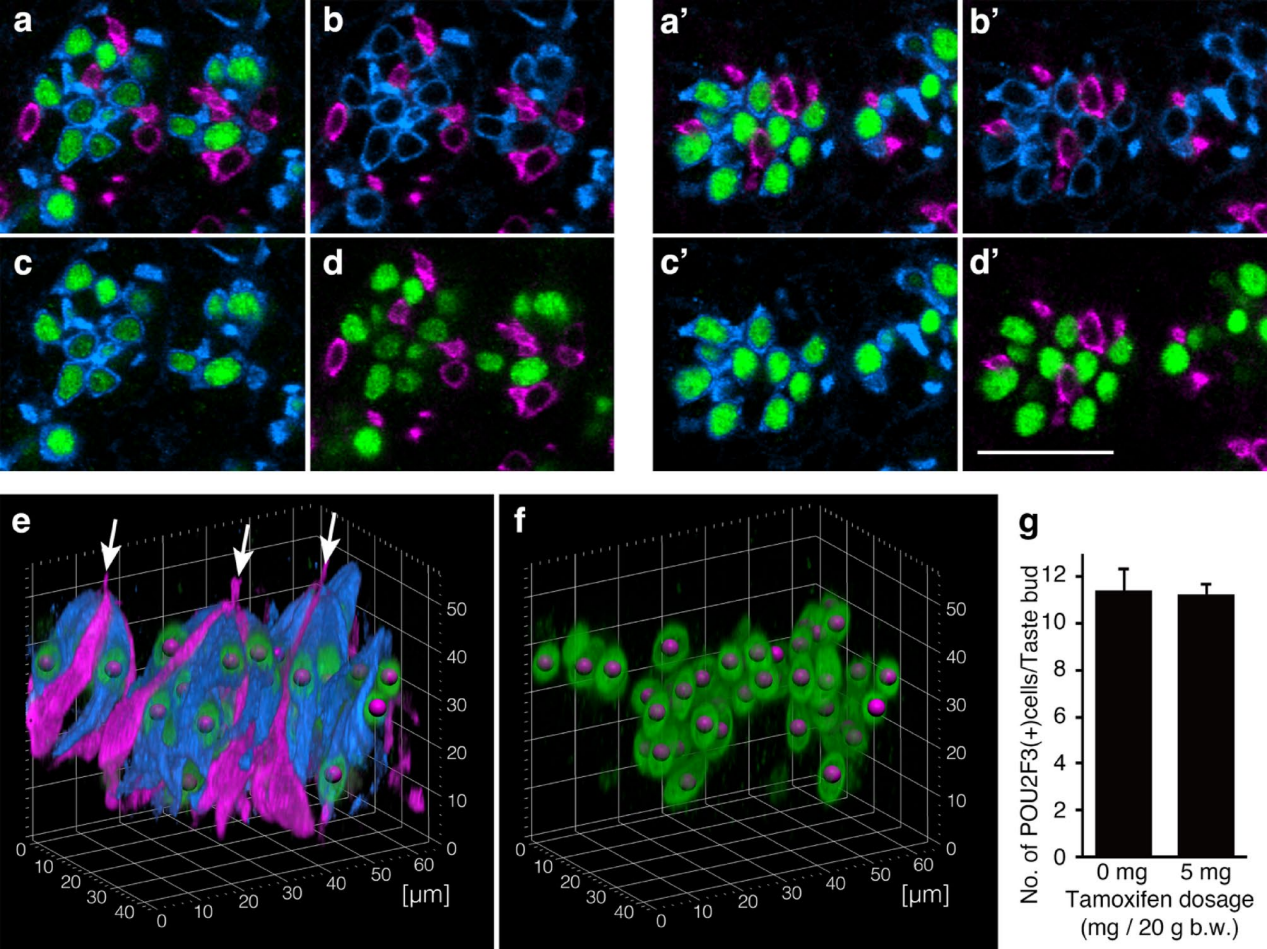
Triple-color immunohistochemistry of the CV for IP3R3 (blue), CA4 (magenta), and POU2F3 (green). (**a**–**d**, **a’**–**d’**) Optical sections from a control (**a**–**b**) and a sample treated with 5 mg tamoxifen (**a’**–**d’**). The scale bar in d’ represents 25 μm and applies to all panels (**a**–**d,** **a’**–**d’**). (**e**, **f**) 3D projection images of the control sample (a-d). Magenta spheres in e and f are spot objects generated by the spot function of Imaris 10.0.1 to detect and count POU2F3(+) nuclei of Type II cells. The arrows in (**e**) indicate the taste pores. (**f**) POU2F3 (green) and the spots extracted from panel (**e**). (**g**) Summary of the number (mean ± SE) of POU2F3(+) cells per taste bud in the CV of control and tamoxifen-treated mice. No significant difference was observed.

To address these issues, we performed triple-color whole-mount immunohistochemistry of the CV epithelium using antibodies against IP3R3 (a Type II cell marker), CA4 (a Type III cell marker), and POU2F3 (a transcription factor specific for Type II cells) one day after tamoxifen injection, following the same schedule as the qPCR analysis (Fig. 1a), and compared the 5 mg dose group with the control group (Fig. 2a–d’). In both groups, the signals for IP3R3 and CA4 were completely segregated (Fig. 2b,b’). POU2F3 was detected exclusively in IP3R3(+) cells and was absent in CA4(+) cells (Fig. 2c,c’,d,d’). In addition, the cells detected by IP3R3 or CA4 antibodies were all fusiform cells and did not include Type I-like shape cells with thin cell processes wrapping neighboring cells (Fig. 2e). These results confirm that the cell type-specific expression pattern of these markers was not altered by tamoxifen injection, even at the 5 mg dose.

Next, we quantified the number of POU2F3(+) cells per taste bud (Fig. 2e–g). No significant difference was observed in the number of POU2F3(+) nuclei per taste bud between the control (11.4 ± 0.9) and the 5 mg dose groups (11.3 ± 0.9; Student’s t-test, *p* = 0.91, *n* = 3 per group) indicating that the number of Type II cells was not altered following tamoxifen injection at this time point.

These results indicate that tamoxifen induces the expression of mature cell type-specific genes in taste buds without altering their specificity or increasing cell numbers.

### Tamoxifen reduces SHH(+) cells but does not alter the number of S-phase cells

**Fig. 3:**
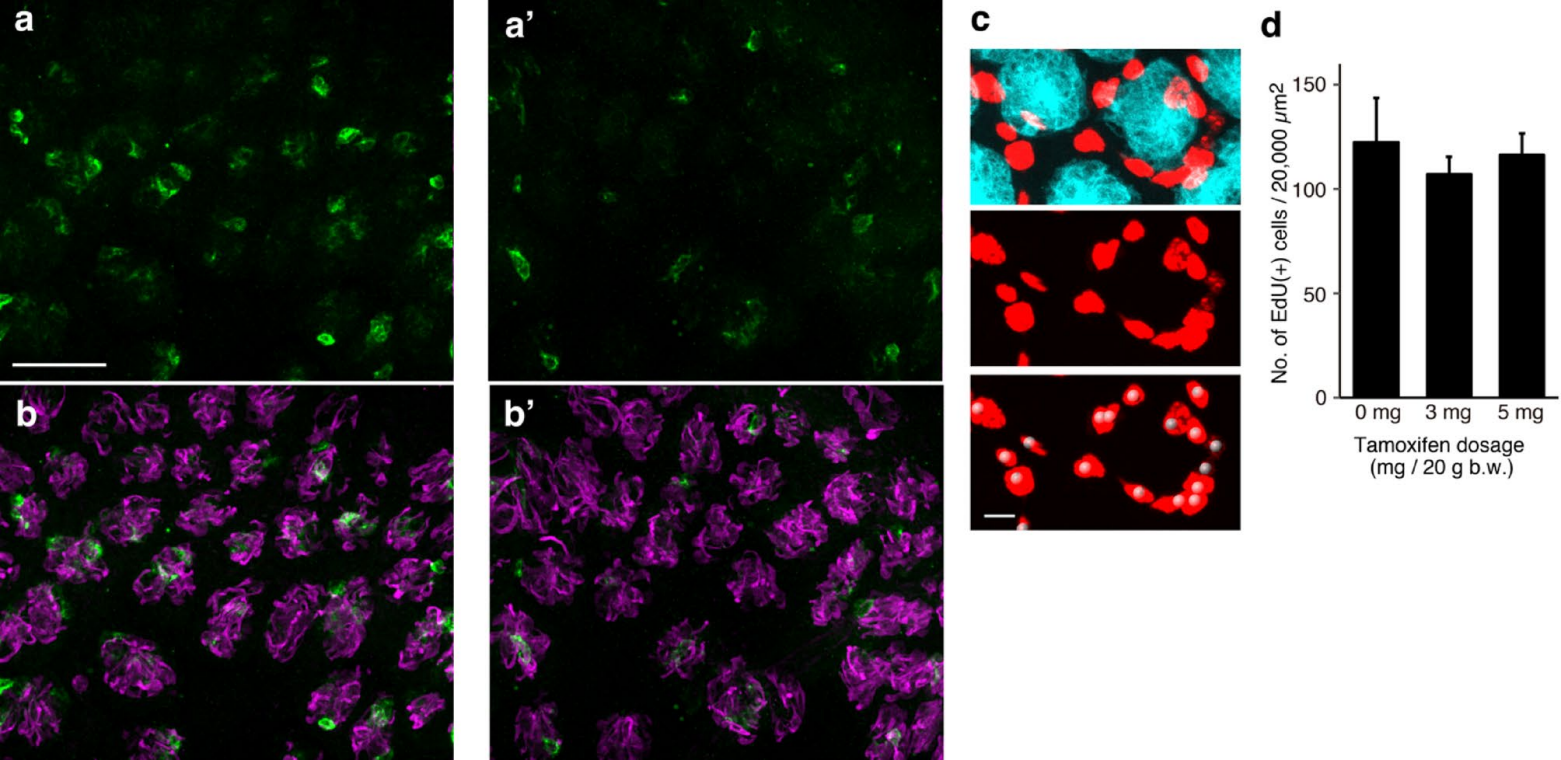
SHH expression and EdU incorporation in the CV epithelium 22 hours after tamoxifen injection. (**a**–**b’**) Whole-mount immunohistochemistry of the CV epithelium detecting SHH (green) and IP3R3 (magenta). 3D projection images obtained from the control (**a**, **b**) and 5 mg tamoxifen-treated (**a’**, **b’**) groups. The number of SHH(+) cells was lower in the 5 mg tamoxifen-treated group than that in the control group. IP3R3 indicates the position of the taste buds, with no differences between the two groups. The scale bar in panel (**a**) represents 50 μm for panels (**a**–**b’**). (**c**, **d**) Number of EdU(+) cells per unit area of the CV epithelium 2 h after EdU injection, administered 22 h after tamoxifen injection. (**c**) Example 3D projection images of KCNQ1(+) cells (cyan) and EdU (red) 2 h post-EdU injection in a mouse injected with 3 mg tamoxifen. The white spheres in the bottom panel indicate the spot objects generated by the spot tool in Imaris to identify EdU(+) nuclei. A portion of the unit area (200 μm × 100 μm) is shown. Scale bar: 20 μm. (**d**) Summary of EdU(+) cells per unit area (mean ± SE). No significant differences were observed.

Following the analysis of mature cell types, we examined Type IV immature cells using whole-mount immunohistochemistry of the CV epithelium to detect SHH expression one day after tamoxifen injection (Fig. 1a) and compared the 5 mg dose group with the control group. SHH expression was markedly decreased in the 5 mg dose group compared to that in the vehicle-injected control group (Fig. 3a–b’). Although SHH was detected steadily, it was difficult to precisely profile and count SHH(+) cells because of the wide range of immunostaining intensities. This may be because SHH is a secretory factor that diffuses into intercellular space. Nonetheless, it was obvious that a single intraperitoneal injection of tamoxifen reduced the number of SHH(+) cells.

SHH is a critical signaling molecule that maintains taste bud structure, ensuring the replenishment of cells from the epithelial cells surrounding the taste buds during turnover^13^. The decrease in SHH signaling may cause a reduction in cell proliferation around the taste buds. Therefore, we evaluated the number of S-phase cells in the circumvallate papilla epithelium. EdU was administered one day after tamoxifen injection, at the same time point as qPCR and histochemical analyses (Fig. 1a) at doses of 0, 3, and 5 mg per 20 g body weight. The number of EdU(+) cells was analyzed 2 hours after EdU injection per unit area of the epithelium (100 μm × 200 μm) (Fig. 3c,d). The analyzed areas were carefully selected to contain nearly the same number of taste buds (22.4 ± 2.5: mean ± SD, *n* = 18). No significant differences were observed in the number of EdU(+) cells (mean ± SE) between the groups (Fig. 3d): 122 ± 21.3 in the 0 mg group, 107 ± 8.4 in the 3 mg group, and116 ± 10.2 in the 5 mg group per 20,000 µm^2^ (ANOVA; F_(2,6)_ = 0.8540, *p* = 0.472). EdU incorporation in the CV epithelium 1 day after tamoxifen injection was comparable to that in the vehicle-injected control group. These results indicate that tamoxifen did not alter the number of S-phase cells around the taste buds one day after injection.

### Tamoxifen reduces the cell supply to taste buds in a dose-dependent manner

As the number of EdU(+) cells in the CV epithelium at 1 day after tamoxifen injection was comparable to that in the vehicle-injected control group (Fig. 3d), we next focused on the impact of tamoxifen on the influx of the EdU(+) cells labeled at this time point and their descendants into taste buds.

**Fig. 4:**
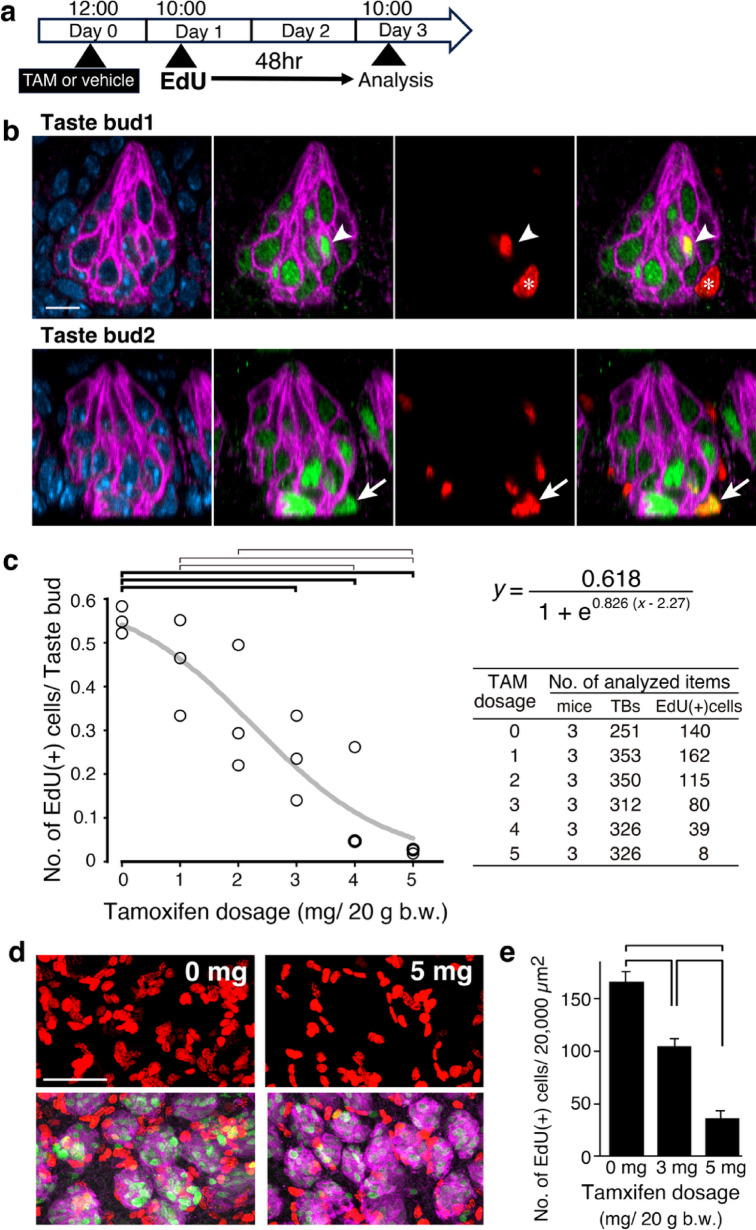
EdU(+) cells observed 48 h after EdU injection, administered 22 h after tamoxifen administration. (**a**) Schedule of tamoxifen and EdU administration and collection of CV epithelium. (**b**) Longitudinal optical sections of two example taste buds from whole-mount immunohistochemistry of a mouse administered 3 mg tamoxifen: KCNQ1 (magenta), PROX1 (green), EdU (red), and Hoechst (blue). Arrowheads in the upper panels indicate EdU signals within the taste bud. The asterisk marks an EdU(+) nucleus of a cell adjacent to the basal portion of the taste bud; this cell is negative for both KCNQ1 and PROX1 and resides outside the taste bud. The EdU(+) cell indicated by the arrow in the bottom panels is a KCNQ1(-) cell sitting in a similar position as the cell marked with an asterisk, but is a PROX1(+) taste bud cell. Scale bar: 10 μm. (**c**) Summary of the dose dependence of the impact of tamoxifen on the influx of EdU(+) cells into taste buds within 48 h after EdU injection. The number of EdU(+) cells per taste bud decreased with increasing tamoxifen doses. The logistic curve fitted to the data is shown in the graph (R^2^ = 0.83). Brackets indicate statistically significant differences (ANOVA; F_(5,12)_ = 12.34, *p* < 0.0005; post-hoc Bonferroni test, *p* < 0.05), and comparisons with vehicle-injected controls (0 mg) are shown in bold. (**d**) 3D projection images of the CV epithelium from whole-mount immunohistochemistry of mice administered 0 and 5 mg tamoxifen: KCNQ1 (magenta), PROX1 (green), and EdU (red). The upper panels show only the EdU signals. The vast majority of EdU(+) cells were epithelial cells outside the taste buds, as the average number of EdU(+) cells regardless of the dose inside the taste buds was less than 0.6 (**c**). Scale bar: 50 μm. (**e**) Summary of the comparison of the number of EdU(+) cells per 20,000 µm^2^ between 0, 3, and 5 mg tamoxifen-injected groups. Brackets indicate statistically significant differences (ANOVA; F_(2,6)_ = 69.2, *p* < 0.0001; post-hoc Bonferroni test, *p* < 0.005).

To address this issue, we designed an experiment, as shown in Fig. 4a. On day 0, tamoxifen (1–5 mg/20 g b.w.) or vehicle (corn oil) was administered intraperitoneally. The following day, EdU (50 mg/20 g b.w.) was injected. 48 h after EdU injection, the CV epithelium was collected, and the cells newly supplied to the taste buds were evaluated by quantifying EdU(+) cells within the taste buds using whole-mount immunohistochemistry.

To identify taste bud cells, we employed PROX1 as a taste bud cell marker in combination with KCNQ1 immunostaining (Fig. 4b). KCNQ1 is a voltage-gated potassium channel widely used as a taste bud cell marker^18^. PROX1 is a homeobox transcription factor whose expression begins with SHH in Type IV cells and persists throughout the cell life^19^. Notably, in the basal region of the taste buds, we observed cells that were PROX1(+) but KCNQ1(-) (Fig. 4b, arrow). These cells indicate that PROX1 is a useful marker for detecting young taste bud cells within 48 h of birth, before the onset of KCNQ1 expression.

Figure 4c summarizes the effect of tamoxifen on the influx of cells into the taste buds. The data were well-fitted to a logistic curve (R^2^ = 0.83), indicating that tamoxifen reduced the number of newly supplied EdU(+) cells to the taste buds in a dose-dependent manner (IC_50_:2.27 mg/ 20 g b.w.). The first significant reduction compared to the vehicle-injected control was observed at 3 mg tamoxifen, and EdU(+) cells were rarely detected in the taste buds at 5 mg tamoxifen. The average frequency of EdU(+) cells per taste bud in the 5 mg dose group was reduced to less than 6% of that in the control group, from 0.55 ± 0.02 to 0.025 ± 0.004 EdU(+) cells per taste bud (mean ± SE).

**Fig. 5:**
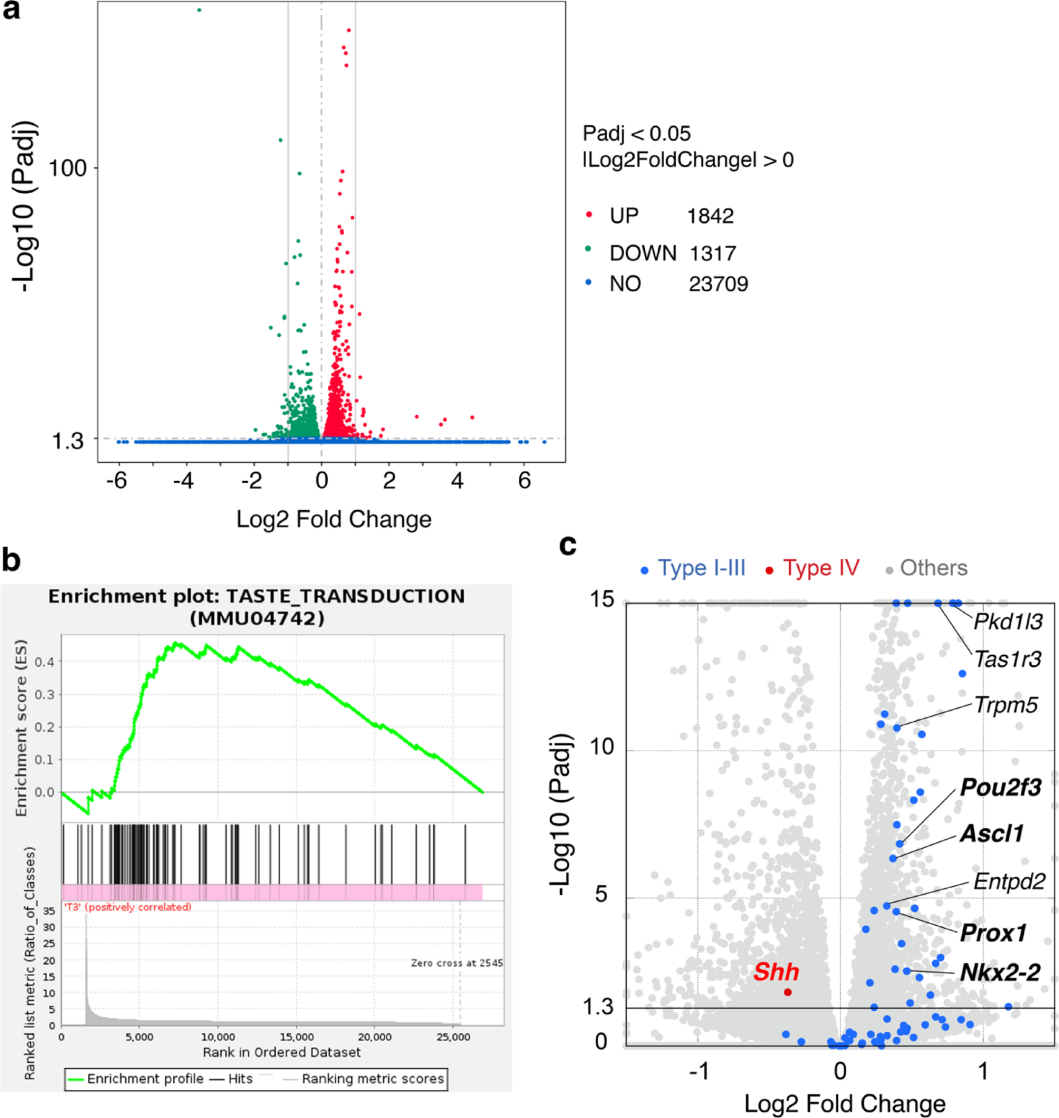
Summary of differentially expressed genes and upregulated KEGG gene set. (**a**) Volcano plot summarizing the relative expression levels of all genes analyzed in the 3 mg tamoxifen-injected group compared to those in the vehicle-injected control group. The horizontal axis represents the Log2Fold Change (Log2FC), and the vertical axis represents -Log10 (adjusted *p*-value (Padj)). Upregulated genes (red), downregulated genes (green), and genes that showed no significant difference in expression levels (blue) (Statistically significance, Padj < 0.05; -Log10 (0.05) ≈ 1.3). The two vertical lines in the graph indicate the boundaries where |Log2FC| = 1, which signifies a 2-fold change. (**b**, **c**) Summary of the TASTE_TRANSDUCTION pathway in 3 mg tamoxifen-injected mice. (**b**) Gene Set Enrichment Analysis (GSEA) plot showing the upregulation of the TASTE_TRANSDUCTOION gene set in the 3 mg group compared to the control group. (**c**) Volcano plot showing the relative expression levels of taste bud cell-specific genes in 3 mg tamoxifen-injected mice compared to those in the control mice. Type I-III specific genes (blue) and Type IV specific *Shh* (red). The transcription factors are shown in bold. The plotted genes are listed in Supplementary Table 1.

The reduction in EdU(+) cells was not restricted to the taste buds but was also observed in the surrounding epithelium (Fig. 4d). Most of the EdU signals shown in Fig. 4d originated from the epithelial cells surrounding the taste buds, considering that the average number of EdU(+) cells per taste bud shown in Fig. 5c was less than 0.6, regardless of tamoxifen dose. The average number (mean ± SE) of EdU(+) cells per 20,000 µm^2^ in the 5 mg dose group was 22% of that in the control group: 187 ± 10 versus 41 ± 7.9 EdU(+) cells per 20,000 µm^2^ (Fig. 4e).

These results demonstrate that tamoxifen injection reduced the replenishment of new cells in the taste buds, along with the reduction of EdU(+) cells in the surrounding epithelium.

### RNA-sequencing analysis of tamoxifen-induced gene expression changes in the CV taste epithelium

To assess the molecular aspects of tamoxifen’s effects on taste buds and the surrounding epithelium in more detail, we performed RNA sequencing (RNA-seq) analysis. Among the tested doses of tamoxifen (0–5 mg), a statistically significant reduction in cell replenishment to taste buds was first observed at 3 mg (3 mg/ 20 g b.w.). As conditional recombination is typically induced with doses of up to 4 mg, we selected 3 mg tamoxifen for RNA-seq analysis in this study.

Tamoxifen or vehicle was intraperitoneally injected into mice, and 22 h after injection, the CV epithelium was collected and analyzed. This schedule is the same as that shown in Fig. 1a. Figure 5a shows a volcano plot of all annotated genes, displaying the log2 fold change (Log2FC; x-axis) in gene expression in tamoxifen-treated mice versus control mice with statistical significance (-log10 adjusted p-value (Padj); y-axis). The overall changes in gene expression were relatively modest, with fewer than 100 genes showing an absolute Log2FC greater than 1. Using a cutoff of |Log2FC| > 1, 60 genes were downregulated, and 28 genes were upregulated in the tamoxifen-treated group. The magnitude of gene expression changes in the taste buds observed by qPCR ranged from 0.6- to 1.4-fold (Fig. 1b), which fell within the overall modest expression changes observed across all annotated genes (Fig. 5a).

### Tamoxifen upregulates mature cell-specific genes but downregulates *Shh* in taste buds

To investigate pathway-level changes in gene expression between tamoxifen- and vehicle-injected groups, we performed gene set enrichment analysis (GSEA) using GO and KEGG pathway annotations. Only one KEGG pathway, TASTE_TRANSDUCTION (MMU04742), showed a statistically significant upregulation, with a normalized enrichment score (NES) of 2.07 and a false discovery rate (FDR) *q*-value of 0.169 (Fig. 5b). Statistical significance was determined using thresholds of FDR *q*-value < 0.25 and nominal *p*-value < 0.05. Figure 5c shows a volcano plot summarizing the changes in the expression of taste bud cell-specific genes. Type I-III cell-type-specific genes were upregulated, consistent with the qPCR results. Notably, transcription factors required for specific cell type differentiation were found in the upregulated genes: *Pou2f3* of Type II cells, and *Ascl1* and *Nkx2-2* of Type III cells. *Prox1*, which is expressed in all taste bud cells, was also upregulated. In contrast, *Shh*, which is specifically expressed in Type IV immature cells, was downregulated, as observed in the qPCR results.

These RNA-seq results demonstrated a coordinated upregulation of virtually all marker genes specific for Type I, II, and III mature cells, confirming and extending the initial findings from qPCR (Fig. 1).

### Transcriptional changes from proliferation to differentiation, accompanied by reduced mitochondrial energy synthesis in the CV taste epithelium

**Fig. 6:**
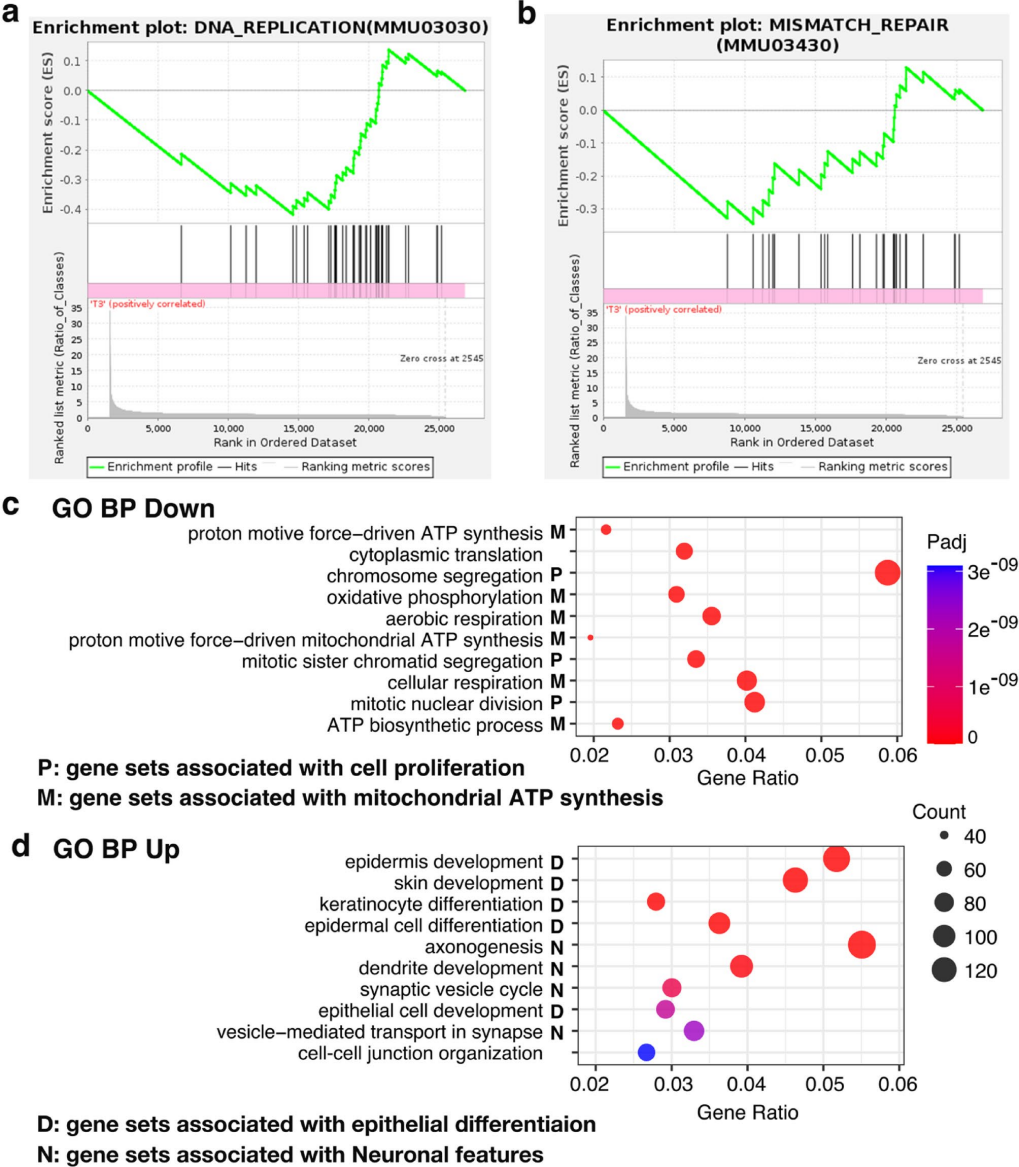
GSEA plots for downregulated KEGG gene sets and GO biological process (BP) enrichment plots for upregulated and downregulated gene sets. (**a**, **b**) GSEA plots of two KEGG gene sets that showed statistically significant decreases: DNA_REPLICATION (**a**) and MISMATCH_REPAIR (**b**). Both gene sets had FDR *q*-values of 0.169. (**c**, **d**) Dot plots of GO BP enrichment analysis. (**c**) Top 10 downregulated gene sets. (**d**) Top 10 upregulated gene sets. The dot size indicates the number of genes in each term, and the color represents the adjusted P value.

In addition to the upregulation of TASTE_TRANSDUCTION, two KEGG pathways showed statistically significant downregulation in the GSEA: DNA_REPLICATION (MMU03030) and MISMATCH_REPAIR (MMU03430) (Fig. 6a,b). The NES values of these pathways were − 1.33 and − 1.27, respectively, suggesting mild to slight downregulation. Both pathways are essential for cell proliferation.

Because the significantly altered pathways revealed by GSEA were limited to one upregulated and two downregulated pathways, we analyzed individual gene sets from the GO Biological Process (BP) category, highlighting the biological functions associated with changes in gene expression. Figure 6c and d show the top ten downregulated and upregulated gene sets ranked by adjusted *p*-value in ascending order.

Among the top 10 downregulated gene sets (Fig. 6c), three pathways were associated with cell proliferation, revealing new gene sets different from the two cell proliferation-related gene sets identified by the GSEA. Six of the remaining pathways among the top 10 were involved in the mitochondrial function of ATP synthesis. Among the top 10 upregulated gene sets (Fig. 6d), five were associated with epithelial cell and keratinocyte differentiation. The remaining four upregulated gene sets were involved in neuronal molecular features. The neuronal elements in the CV epithelium may represent gene expression in taste bud cells which generate action potentials by receiving taste stimuli and subsequently release neurotransmitters.

**Fig. 7:**
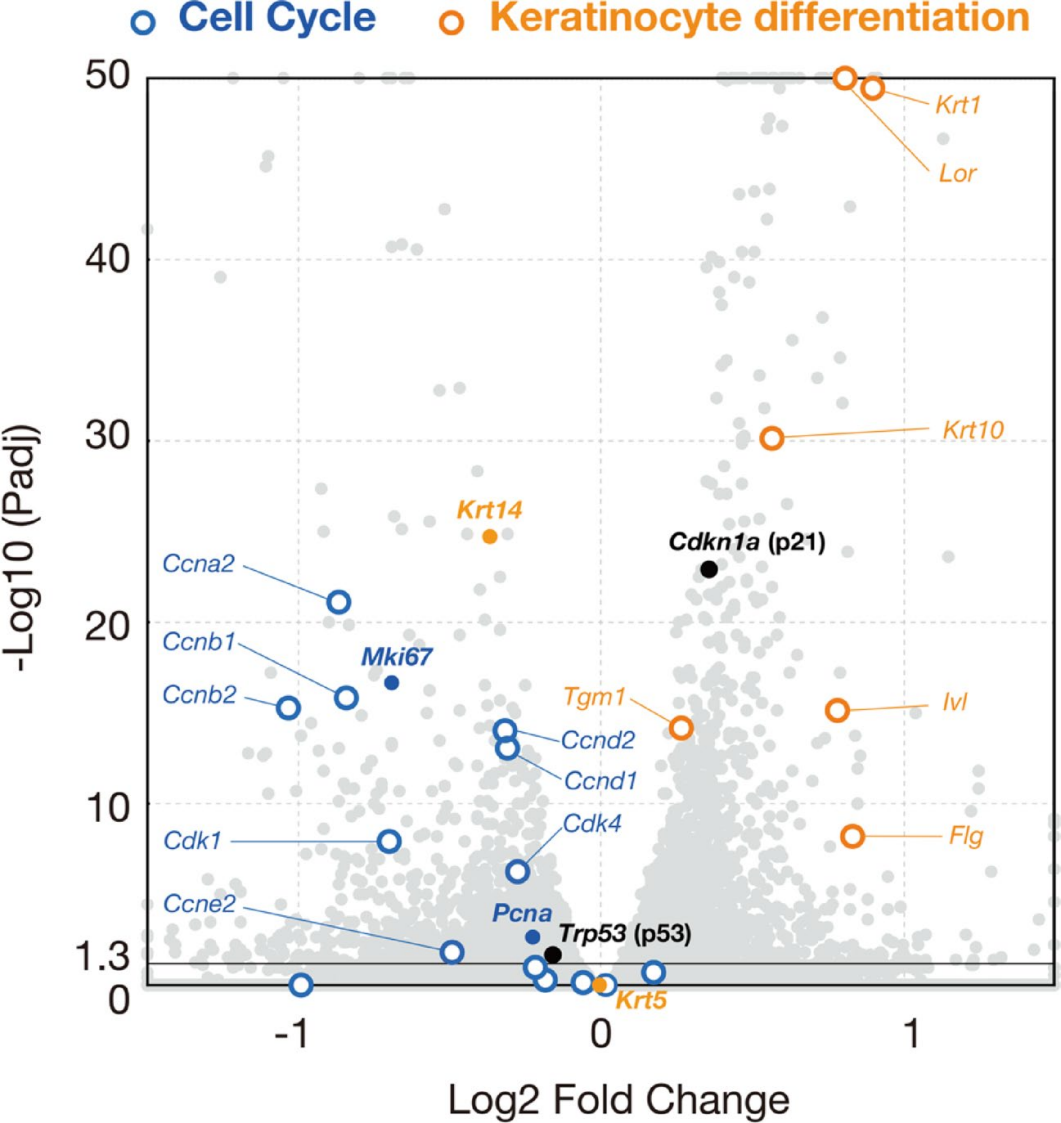
Volcano plot showing the relative expression levels of genes associated with keratinocyte differentiation and the cell cycle. The blue circles represent cyclins and cyclin-dependent kinases that promote cell cycle progression. Blue dots indicate the proliferation markers *Mki67* and *Pcna*. The black dots indicate *Cdkn1a* (p21), a cyclin-dependent kinase inhibitor that suppresses cell cycle progression, and *Trp53* (p53), a transcription factor that induces *Cdkn1a* expression. The orange circles represent keratinocyte-related genes, and orange dots indicate keratin genes expressed in basal immature cells. The plotted genes are listed in Supplementary Table 2.

These changes in pathways prompted us to examine the expression of individual genes related to keratinocyte differentiation and the cell cycle (Fig. 7). Genes required for keratinocyte differentiation, such as *Krt10*,* Inv*, and *Flg*, were upregulated, whereas the immature epithelial cell-specific gene, *Krt14*, was downregulated. For cell proliferation-related genes, the proliferation markers *Mki67* and *Pcna*, as well as most cyclins and cyclin-dependent kinases, were downregulated. In contrast, the cyclin-dependent kinase inhibitor *Cdkn1a* (p21) was upregulated, whereas *Trp53* (p53), a regulator of p21, was slightly downregulated.

Taken together, these transcriptomic changes indicate that tamoxifen administration shifts the transcriptional program from proliferation to differentiation, accompanied by reduced mitochondrial energy synthesis in the CV taste epithelium.

### Tamoxifen-induced expression changes in transcription factors regulating the balance between proliferation and differentiation

Next, we sought to identify the transcription factors involved in the tamoxifen-induced upregulation of differentiation-related gene expression and suppression of epithelial cell proliferation. Table 1 summarizes the five most significantly upregulated and downregulated transcription factors. Three of these factors have been implicated in the regulation of epithelial cell proliferation and differentiation.

**Table 1:** Top 5 upregulated and downregulated transcription factors.

	Gene	Log2FC	Adjusted *p*-value	Mean normalized readcount		Gene description
				Corn oil	Tamoxifen	
Up	*Zbtb16*	1.29	5.59 × 10^− 7^	132	322	Zinc finger and BTB domain containing 16
	*Fosb*	1.25	1.36 × 10^− 12^	266	631	FBJ osteosarcoma oncogene B
	*Fos*	1.13	2.06 × 10^− 47^	1430	3133	FBJ osteosarcoma oncogene B
	*Tsc22d3*	0.79	5.67 × 10^− 18^	1460	2524	TSC22 domain family, member 3
	*Prdm1*	0.74	7.47 × 10^− 40^	5914	3524	PR domain containing 1, with ZNF domain
Down	*Dbp*	-1.26	1.03 × 10-^39^	2100	877	D site albumin promoter binding protein
	*Spi1*	-0.89	4.78 × 10^− 3^	242	130	Spleen focus forming virus (SFFV) proviral integration oncogene
	*Atf3*	-0.89	9.07 × 10^− 5^	516	279	Activating transcription factor 3
	*Foxm1*	-0.8	2.88 × 10^− 10^	1207	695	Forkhead box M1
	*Zfp202*	-0.71	2.00 × 10^− 2^	246	151	Zinc finger protein 202

Transcription factors are listed in descending order of the absolute values of log2FC.

Among the upregulated factors, *Fos* and *Fosb* belong to the activator protein-1 (AP-1) family, which is known to play an essential role in epithelial cell differentiation^20^. Conversely, *Foxm1*, one of the downregulated factors, is known to maintain the proliferation of stem and progenitor cells^21^, and its downregulation is involved in keratinocyte differentiation^22^. Other transcription factors showed fewer direct connections to the regulation of epithelial differentiation, which may reflect various cellular responses to tamoxifen that were not identified in the RNA-seq analysis. The potential regulatory roles and mechanistic implications of AP-1 and *Foxm1* are presented in the Discussion section.

## Discussion

In this study, we demonstrated that tamoxifen suppresses cell replenishment in the taste buds in a dose-dependent manner. We also found that a single intraperitoneal administration of tamoxifen at a commonly used dose (3 mg/20 g b.w.) for the tamoxifen-inducible Cre-loxP system induced substantial changes in gene expression. Within the taste buds, marker genes representing all taste cell types were coordinately upregulated, whereas *Shh* expression was reduced in the immature precursor cells. In the surrounding epithelium, genes associated with epithelial and keratinocyte differentiation were upregulated, whereas *Krt14* expression was downregulated in basal immature cells. These findings indicate that tamoxifen triggers a transcriptional switch from proliferation to differentiation both within taste buds and in the surrounding epithelium, highlighting that tamoxifen itself may represent a confounding factor in taste bud research using the tamoxifen-inducible Cre-loxP system. AP-1 and *Foxm1* emerged as potential key regulators driving tamoxifen-induced transcriptional changes in the CV taste epithelium.

### Induction of gene expression within taste buds

RNA-seq analysis demonstrated that tamoxifen broadly induced gene expression in taste buds, whereas *Shh*, the only gene known to be specifically expressed in immature precursor cells, was downregulated. The upregulated genes included key transcription factors that drive the differentiation of specific cell types: *Pou2f3* for Type II cells, and *AsclI* and *Nkx2-2* for Type III cells^23–25^. Although no transcription factor responsible for Type I cell differentiation has yet been identified, Type I cells are also likely to be in a similar state, as multiple Type I cell-specific genes, including *Entpd2* and *Kcnj1*, are upregulated. Furthermore, *Prox1* was upregulated by tamoxifen; its expression begins with *Shh* at the birth of taste bud cells and persists throughout their lifespan^19^. These results suggest that tamoxifen drives transcriptional changes that promote the functional differentiation of taste bud cells via fundamental regulatory mechanisms shared by all taste cell types, rather than through cell type-specific mechanisms.

Such fundamental mechanisms have so far been demonstrated for taste bud development and maintenance involving *Shh* and *R-spondin* signaling^12,26–28^: each of these signaling pathways alone can induce taste bud formation or maintenance, respectively. Both pathways not only induce taste cell differentiation but also support cell replenishment in the taste buds. However, because tamoxifen reduces cell replenishment, these pathways are less likely to be the primary drivers of tamoxifen-induced transcriptional changes. As an alternative candidate mechanism, AP-1 has been implicated in taste bud maintenance based on the conditional knockout of *Fos* in *Krt14*-expressing basal cells, which include taste bud stem cells^29^. Nonetheless, the role of *Fos* in differentiating taste cells and the consequences of its upregulation remain unclear. In the present study, RNA-seq analysis revealed the upregulation of AP-1 components together with taste bud-specific genes in the CV taste epithelium. Further functional studies are required to determine whether AP-1 plays a critical role in tamoxifen-induced transcriptional changes that lead to taste bud cell differentiation.

### Tamoxifen dose-dependent reduction in EdU(+) cells in the CV epithelium and cell replenishment in taste buds

RNA-seq revealed the downregulation of cell proliferation-related gene sets at 22 h after tamoxifen injection, whereas EdU injection at this time demonstrated a tamoxifen dose-dependent reduction in EdU(+) cells within and around taste buds 48 h later. However, the frequency of S-phase cells at 22 h was comparable between the tamoxifen- and vehicle-injected groups, indicating that the S-phase of the cell cycle was maintained normally at this time point despite the transcriptional changes. Therefore, it is plausible that cell proliferation is functionally downregulated during the subsequent 48 h, leading to reduced cell replenishment in the taste buds and fewer EdU(+) cells in the surrounding epithelium.

RNA-seq also revealed impaired mitochondrial respiration in the CV epithelium, which is a typical response to tamoxifen caused by the inhibition of protein kinase C^15^. Mitochondrial dysfunction may contribute to the suppression of cell proliferation by limiting ATP availability for cell cycle progression.

Among the various effects of tamoxifen^15^, the induction of apoptosis could also contribute to fewer EdU(+) cells 48 h after injection. However, no apoptosis-related gene sets were identified among the most significantly altered pathways in the RNA-seq analysis 22 h after tamoxifen injection. When all gene sets with an adjusted p-value < 0.05 were considered, several apoptosis-associated gene sets were identified; however, they ranked lower and were distributed across both upregulated and downregulated categories (Supplementary Table 3). Moreover, no significant upregulation of pro-apoptotic genes was observed; however, a few pro-apoptotic genes, such as *Bak1* and *Trp53*, showed statistically significant decreases after tamoxifen injection (Supplementary Table 4). These results are more consistent with a balanced regulation of apoptosis rather than a shift towards its induction at 22 h. Other cell death-related gene sets, such as those related to necrosis and ferroptosis, were not significantly altered. To clarify the involvement of cell death in the reduction of EdU(+) cells and taste bud cell replenishment by tamoxifen, further RNA and histochemical analyses of cell death at later stages are required.

In the CV epithelium, when different tamoxifen doses were compared, the number of EdU(+) cells was lower at higher tamoxifen doses 48 h after EdU injection, with no differences observed 2 h after injection. This finding likely reflects a tamoxifen dose-dependent decrease in the proliferation rate of EdU(+) cells. Consistent with this interpretation, the RNA-seq data demonstrated reduced expression of cell proliferation–related genes at 22 h, as described above. However, focusing on the 5 mg tamoxifen dose, the decrease in EdU(+) cell density observed from 2 to 48 h after EdU injection suggests that EdU-negative cells, which were not in the S-phase at the time of labeling, likely subsequently proliferated following a transient suppression of cell division. This proliferative activity may have occurred after the proliferation suppression phase detected by RNA-seq. Further analyses, including the use of two different thymidine analogs, are required to better understand cell proliferation dynamics after tamoxifen treatment.

Within taste buds, the reduction in cell supply is most likely caused by a general decrease in new cell generation from bipotential stem cells, given the accompanying significant reduction in EdU(+) cells in the surrounding epithelium, as described above. However, it remains possible that tamoxifen alters the balance of cell differentiation between taste bud and surrounding epithelial cells, generating more surrounding cells.

### Regulatory mechanisms of transcriptional changes from proliferation to differentiation in the taste epithelium

Among the transcription factors, AP-1 and *Foxm1* appear to be directly involved in tamoxifen-induced transcriptional changes in the taste epithelium. *Fos* and *Fosb*, members of the AP-1 family, were among the upregulated transcription factors. Although AP-1 was originally identified as a stress-inducible factor, particularly in response to reactive oxygen species (ROS), it also plays an essential role in normal epithelial and keratinocyte differentiation^20^. In this context, AP-1 contributes to the barrier function of the epithelium against various types of cellular stress. Because tamoxifen induces cellular stress, it likely activates AP-1 expression in the taste epithelium, thereby promoting differentiation^15^. Taken together, AP-1 may mediate tamoxifen-induced transcriptional changes towards differentiation, both within taste buds and in the surrounding epithelium.

Among the downregulated factors, *Foxm1*, a member of the forkhead superfamily, is a well-characterized proliferation-associated transcription factor that drives cell cycle progression and mitosis in various tissues^30^. *Foxm1* promotes cell cycle progression by repressing *Cdkn1a* (p21)^31^ and inducing CDK1 expression^32^, and is downregulated during keratinocyte differentiation^22^. In the CV epithelium, tamoxifen upregulated p21 and downregulated cyclins and cyclin-dependent kinases, whereas *Foxm1* was downregulated concurrently with the transcriptional activation of the differentiation programs. Given that *Foxm1* is involved in maintaining the pluripotency and self-renewal capacity of stem cells^30^, it may serve as a critical regulator of taste epithelial homeostasis.

Furthermore, *Foxm1* represses AP-1 expression by modulating chromatin accessibility and transcriptional changes^33^. Thus, the downregulation of *Foxm1* may directly induce AP-1 expression. *Foxm1* is also known to upregulate ROS scavengers, such as catalase, superoxide dismutase 2, glutathione peroxidase 2, and PRDX, which neutralize ROS^22^. Consequently, *Foxm1* downregulation can elevate ROS levels, which may in turn induce AP-1 expression. This indirect mechanism of AP-1 regulation via ROS is likely to act in concert with the direct effect of *Foxm1* on AP-1 expression. The interplay among *Foxm1*, ROS, and AP-1 suggests a complex regulatory network with important implications for cell proliferation, differentiation, and stress responses in the CV epithelium.

### Insights and perspectives on mitigating direct effects of tamoxifen on the CV epithelium

The tamoxifen effects observed in this study, such as suppression of the cell cycle and mitochondrial respiration, are known to occur via ER-independent mechanisms^15^. However, in the CV epithelium, Dahir et al.^34^. reported that ERα and GPER1 are expressed in taste buds regardless of sex, with GPER1 largely restricted to Type II cells. They suggested that the expression of Trpm5, a Type II cell-specific signaling molecule in taste buds, may be directly induced by estradiol in female mice. Therefore, the ER-mediated mechanism of tamoxifen may contribute to the effects on CV epithelium, particularly in the alteration of taste cell-specific gene expression. Further studies focusing on ERs are necessary to elucidate the molecular pathways that mediate the effects of tamoxifen on CV epithelium.

In this study, a single intraperitoneal injection was used to precisely control the dosage and timing of administration, which is critical for the evaluation of the direct effects of tamoxifen. This method allowed estimation of the half-maximal inhibitory concentration (IC_50_) of tamoxifen, which was estimated at 2.27 mg per 20 g body weight for suppression of the cell supply to taste buds. This quantitative measure provides a useful benchmark for dosing in future experiments aimed at assessing taste bud cell dynamics by using tamoxifen-inducible recombination systems. Indeed, around 2.27 mg/ 20 g b.w. might be a critical dose to avoid the significant inhibitory effect of tamoxifen on cell replenishment in taste buds, as one study reported that no statistically significant difference was observed in the number of Type II cells even after a 10-day consecutive daily single intraperitoneal injection of 2 mg/ 20 g b.w. tamoxifen, compared to the non-tamoxifen-treated groups^35^.

In addition to intraperitoneal injections, oral gavage and chow diets are often used for tamoxifen administration. Regarding chow diets, according to the manufacturer of the tamoxifen-containing chow, the diet contains 400 mg/ kg tamoxifen citrate and provides an estimated daily intake of approximately 0.8 mg tamoxifen/ 20 g body weight (Inotiv, Inc. The tamoxifen diet (Product No. 130860), Technical Data Sheet. Available at:https://www.inotiv.com (accessed 16 November 2025)). This dose appears sufficiently low to avoid the direct effects of tamoxifen, and this type of chow diet has been used in taste bud research^13,36^. However, differences in the direct effects of tamoxifen on the taste epithelium between administration routes have not been directly assessed. Further systematic research is needed to evaluate the differences and establish standardized protocols.

## Conclusion

Our study demonstrates that tamoxifen, at doses commonly used for the Cre-loxP system, significantly alters cellular dynamics in the CV taste epithelium by suppressing cell replenishment in the taste bud and promoting transcriptional changes towards differentiation. Because tamoxifen itself modifies transcriptional programs and cellular homeostasis, its use in taste bud research should be carefully considered as a potential confounding factor. The coordinated transcriptional switch from proliferation to differentiation was accompanied by the downregulation of *Foxm1* and upregulation of AP-1, suggesting a regulatory network that may involve direct and indirect interactions mediated by ROS. Further studies are required to clarify the molecular mechanisms underlying *the Foxm1*-AP-1 interplay and the role of oxidative stress in taste epithelial homeostasis. Such insights could provide a foundation for developing therapeutic strategies aimed at enhancing taste receptor function and promoting recovery from taste disorders.

## Materials and methods

### Experimental animals

Wild-type C57BL/6J male mice were purchased from Japan SLC (Shizuoka, Japan). Male mice (8–12 weeks of age; 24.8 ± 2.1 g of body weight (mean ± SD, *n* = 80)) were used in this study to avoid the potential confounding effects of the estrogen cycle in female mice. Mice were housed under a 12-hour light/dark cycle (lights on from 7:00 to 19:00) with ad libitum access to food and water. All experimental procedures were approved by the Kagoshima University Animal Experiment Committee and were performed in accordance with the guidelines and regulations of the Committee. All methods are reported in accordance with ARRIVE guidelines (https://arriveguidelines.org).

### Tissue preparations

The mice were euthanized solely by cervical dislocation to minimize potential metabolic alterations in the taste epithelium, in accordance with the guidelines approved by the Kagoshima University Animal Committee, and the tissues were harvested. Taste epithelia from the CV were obtained following enzymatic treatment, as previously described^37^. Briefly, Ringer’s solution containing 2.5 mg/ml collagenase type IV (Worthington Biochemical, Lakewood, NJ) and 2 mg/ml elastase (Worthington Biochemical) was injected under the epithelium, and the tissues were incubated at 37 °C for 30 min. The epithelium was then peeled off using forceps in PBS, fixed with 4% paraformaldehyde (PFA) in PBS for 1 h at room temperature, and processed for immunohistochemistry.

### Whole-mount immunohistochemistry

Whole-mount immunohistochemistry was performed as previously described with some modifications^37^. The peeled epithelium was incubated in 10 mM citrate (pH 6.0) at 100 °C for 10 min (to detect PROX1), 105 °C for 1 min (to detect IP3R3, CA4, and POU2F3), or 105 °C for 3 min (to detect SHH and IP3R3) using an electric pressure cooker (EL-MB30, Zojirushi Corp., Osaka, Japan) for antigen retrieval. The tissues were washed 3 times with TBST (Tris-buffered saline [TBS] containing 0.05% Tween 20) for 15 min each. The tissues were incubated in TBSB (TBS containing 5% normal donkey serum and 0.3% Triton X-100) for 1 h at room temperature and incubated with primary antibodies diluted in TBSB (TBS containing 2.5% normal donkey serum and 0.1% Triton X-100) overnight at 4 °C (Supplementary Table 5). The tissues were washed three times with TBST for 15 min each and incubated overnight at 4 °C (Supplementary Table 6) with secondary antibodies in TBSB containing Hoechst 33342 (Invitrogen, Thermo Fisher Scientific, MA, USA). The tissues were washed three times with TBST for 15 min each. Finally, the tissues were rinsed with TE (10 mM Tris-HCl (pH 8.0), 1 mM EDTA), mounted, and cover-slipped with RapiClear^®^ 1.49 (SunJin Lab, Hsinchu, Taiwan).

### Image processing

Immunofluorescence was imaged using a Zeiss LSM900 confocal microscope with a 40×/1.4 oil-immersion lens. A series of optical sections was acquired at 0.7 μm intervals from the bottom to the apical portion of the taste bud. Three-dimensional images were reconstructed from the z-stack and resliced using Imaris software version 10.0.1 (Bitplane AG, Zürich, Switzerland). Brightness and contrast were adjusted using Imaris and/or Adobe Photoshop (Adobe Systems, San Jose, CA, USA).

### Statistical analysis

Statistical software KaleidaGraph version 5.0.6 (Synergy Software, Reading, PA, USA) and R version 4.5.0 (https://www.r-project.org/) were used for statistical analysis.

Statistical comparisons between the two groups were performed using the Student’s *t*-test. Equal variance was confirmed before the *t*-test using the *F*-test for all two-group comparisons. For multiple group comparisons, ANOVA was performed, followed by post-hoc testing using Bonferroni correction. The threshold for statistical significance was set at α = 0.05.

### Tamoxifen injection

Tamoxifen (Sigma-Aldrich, Merck KGaA, St. Louis, MO, USA) was dissolved in corn oil (Sigma-Aldrich) and administered via a single intraperitoneal injection at a dose of 1–5 mg/20 g body weight (b.w.) (150 µL /20 g b.w.) at 12:00 p.m. The control mice received only the vehicle (corn oil).

### RNA analysis

Total RNA was extracted from the CV epithelium using the RNeasy Plus Mini Kit (QIAGEN, Venlo, The Netherlands) according to the manufacturer’s instructions.

#### Quantitative real-time PCR (qPCR)

The CV epithelium from vehicle- or tamoxifen-administered mice (3 and 5 mg/20 g b.w.) was used for analysis. Total RNA was extracted from independent biological samples (*n* = 6 per group). Complementary DNA (cDNA) was synthesized from total RNA using ReverTra Ace^®^ qPCR RT Master Mix with gDNA Remover (TOYOBO, Osaka, Japan) according to the manufacturer’s instructions. qPCR was performed using the Thunderbird Next SYBR qPCR Mix (Toyobo) on a CFX Duet (Bio-Rad, CA, USA). The PCR assay was performed in five replicates for each sample. Primer sequences used for analysis were listed in Supplementary Table 7. Gene expression levels were normalized to the housekeeping gene (*β-actin*), and relative expression levels were quantified using the 2^-ΔΔCt^ method.

#### RNA-sequencing

CV epithelium from vehicle- or tamoxifen-treated mice (3 mg/20 g b.w.) was used for the analysis. RNA was extracted from 8 control mice and 7 mice treated with tamoxifen at a dose of 3 mg/20 g b.w., and two libraries were constructed for each group. Total RNA samples were submitted to Novogene (Beijing, China) for conventional RNA sequencing. Library preparation, including directional (strand-specific) library construction for eukaryotic mRNA sequencing, sequencing, and initial data processing were performed by Novogene according to their standard protocols. Sequencing was carried out on the Illumina NovaSeq X Plus platform with paired-end 150 bp reads, generating approximately 9 Gb of data per sample (approximately 30 million reads). RNA-seq reads were aligned to the mouse reference genome mm39 using HISAT2 v2.2.1^38^. Our RNA-seq data were deposited in the DDBJ BioProject database under accession number PRJDB35951.

Differential gene expression analysis was performed using DESeq2 version 1.42.0 with adjusted p-values (Padj) < 0.05^39^. The results were visualized using volcano plots, where log2 Fold Changes were plotted against the -log10(Padj).

Pathway enrichment analyses including Gene Ontology (GO) (http://www.geneontology.org/) and Kyoto Encyclopedia of Genes and Genomes (KEGG)^40^, were performed using ClusterProfiler^41^ to identify biological processes and pathways significantly enriched among differentially expressed genes. Significantly altered pathways identified by enrichment analyses were visualized using dot plots to show the degree of enrichment and significance. Gene Set Enrichment Analysis (GSEA) analysis was conducted using GSEA software version 4.3.2 (Broad Institute, MA, USA) to assess the enrichment of predefined gene sets. For GSEA, the false discovery rate (FDR) threshold was set at 0.25 for significance.

### Expression analysis of taste cell type markers and quantification of POU2F3(+) cells

To evaluate the impact of tamoxifen on taste bud cell differentiation, the CV epithelium was harvested 22 h after tamoxifen administration (0 or 5 mg/20 g b.w.), at the same time point as that used for qPCR analysis, and whole-mount immunohistochemistry was performed to detect POU2F3, IP3R3, CA4, and SHH. Among these, POU2F3(+) cells were used for the quantification of Type II cells because the simple ovoid shape of POU2F3(+) nuclei allowed their systematic detection and quantification with the Spot tool in Imaris software, using a diameter threshold of 4.4 μm. SHH was detected with IP3R3 to visualize the location of the taste buds.

### EdU administration and quantification of EdU(+) cells

EdU solution (50 mg/kg body weight) was administered via a single intraperitoneal injection at 10:00 a.m., 22 h after tamoxifen injection. The CV epithelium was collected and used for the following quantifications: (1) S-phase cells, (2) cell supply to the taste buds, and (3) cell replenishment in the circumvallate epithelium. EdU was detected using Click-iT^®^ Plus EdU Imaging Kit (Thermo Fisher Scientific) according to the manufacturer’s instructions.

#### Quantification of S-phase cells in taste epithelium

The CV epithelium from vehicle or tamoxifen-administered mice (3 and 5 mg/20 g b.w.) was used for analysis.

To analyze S-phase cells, the CV epithelium was collected 2 hours after EdU injection. EdU(+) cells were counted in 200 μm × 100 μm (20,000 µm^2^) rectangular regions within the flat epithelial areas of the CV trench walls, which were selected to include a similar number of taste buds among the counting samples using the Imaris software. Two regions were selected from each mouse for analysis. To detect individual EdU(+) nuclei, the Spot tool in Imaris was used with a diameter threshold of 4.4 μm. This value was empirically determined to reliably identify one spot per EdU-labeled nucleus. Nuclei on one long and one short edge of each counting region analyzed were systematically excluded to avoid double-counting. The number of S-phase cells was expressed as the number of spots detected per 20,000 μm².

#### Analysis of dose dependence of the impact of tamoxifen on cell supply to taste buds

The CV epithelium from vehicle- or tamoxifen-administered mice (0–5 mg/20 g b.w.) was collected at 10:00 a.m., 48 h after EdU injection. After immunohistochemistry for KCNQ1 and PROX1, EdU was detected. Two trench walls of the CV from each mouse were analyzed. The number of taste buds and EdU(+) cells was counted. The mean number of EdU(+) cells per taste bud was calculated and plotted. The logistic curve was modeled using the equation *y* = *L/(1 + e*^*k(x−x0)*^*)* with KaleidaGraph software.

#### Analysis of cell replenishment in the circumvallate papillae

EdU(+) cells in the taste epithelium were quantified 48 h after EdU injection using the same procedure as shown in the Quantification of S-phase cells in taste epithelium, in counting areas of 150 μm × 150 μm. The number of EdU(+) cells was expressed as the number of spots per 20,000 μm².

### Experimental design for analysis of the impact of Tamoxifen on taste epithelium

We used tamoxifen ranging from 1 to 5 mg/20 g b.w. and 3 mg/20 g b.w. for the RNA-seq analysis. This dose aligns with the typical tamoxifen amounts used for taste bud studies (up to 4 mg/20 g b.w.), ensuring that our results are relevant and directly applicable to current studies on taste buds using the tamoxifen-inducible Cre-loxP system.

RNA analysis, including qPCR and RNA-seq, was performed 22 h after tamoxifen injection. According to a recent pharmacokinetic study^42^, tamoxifen was detected in the serum 24 h after a single intraperitoneal injection (2 mg/20 g b.w.), while its level rapidly decreased with a half-life of 4.8 h after 8 h post-injection. Its active metabolite, 4-hydroxytamoxifen, also remained detectable in the serum with a half-life of 18.2 h after 8 h of its peak level post injection. Thus, the gene expression changes detected at 22 h after injection are likely to represent direct transcriptional responses to residual tamoxifen and its metabolites rather than secondary downstream effects.

Cell replenishment was analyzed 48 h after EdU injection, which was performed 22 h after tamoxifen administration, the same time point when RNA was analyzed. The turnover rate of the taste epithelium is rapid. Taste bud cells have an average lifespan of 10–14 days^43,44^, with a half-life of about 11 days^45^, whereas the surrounding epithelial cells turnover even faster, with a half-life of about 2 days. The 48-hour interval corresponds to the period during which the proportion of labeled cells among *Shh*(+) Type IV cells reaches its peak level after thymidine analog administration^46^. *Shh*(+) Type IV cells are the first taste bud cells derived from the epithelium surrounding the taste buds and are postmitotic. To minimize potential confounding factors, such as the duration of tamoxifen efficacy and tissue recovery time, we reasoned that EdU(+) cells in taste buds should be analyzed as soon as possible after administration. Thus, the analysis was performed 48 h after EdU injection to ensure reliable detection of EdU in taste buds and minimize the time interval.

## Supplementary Information

Below is the link to the electronic supplementary material.

Supplementary Material 1

## Data Availability

All data presented in this article were generated or analyzed during this investigation. The RNA-seq data were deposited in the DDBJ BioProject database under accession number PRJDB35951. Further inquiries can be directed to the corresponding author(s).
